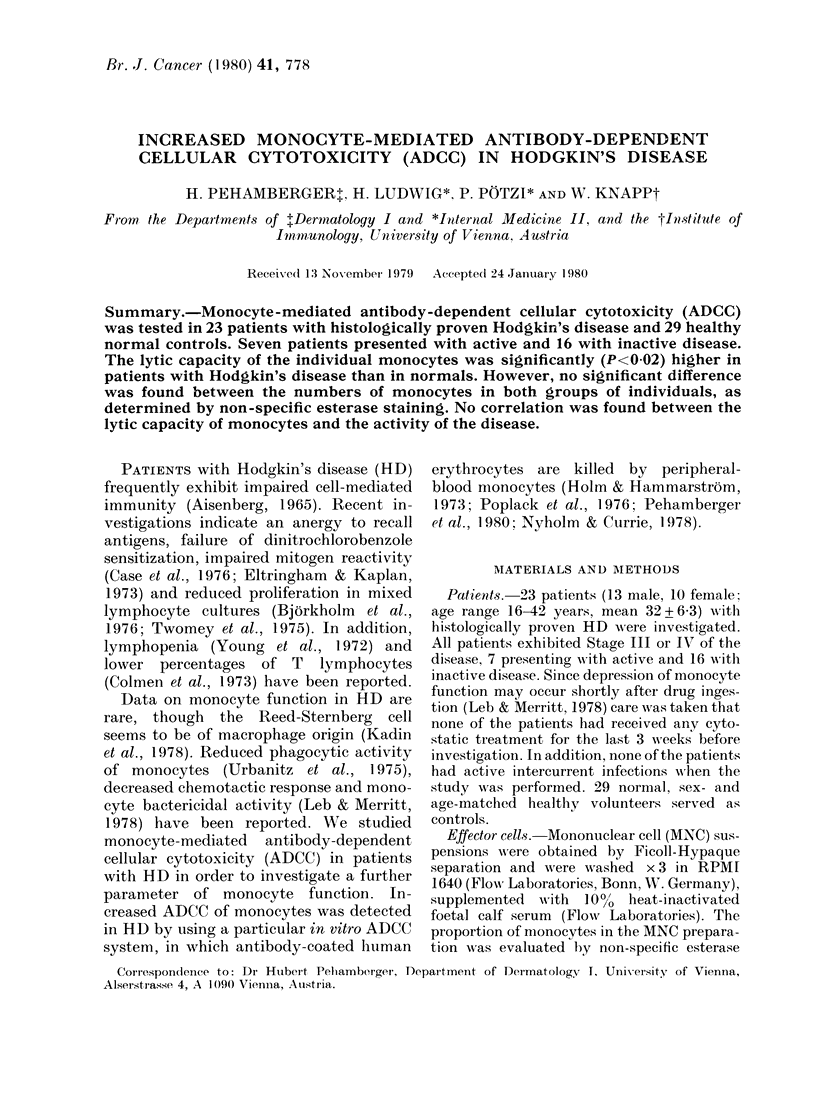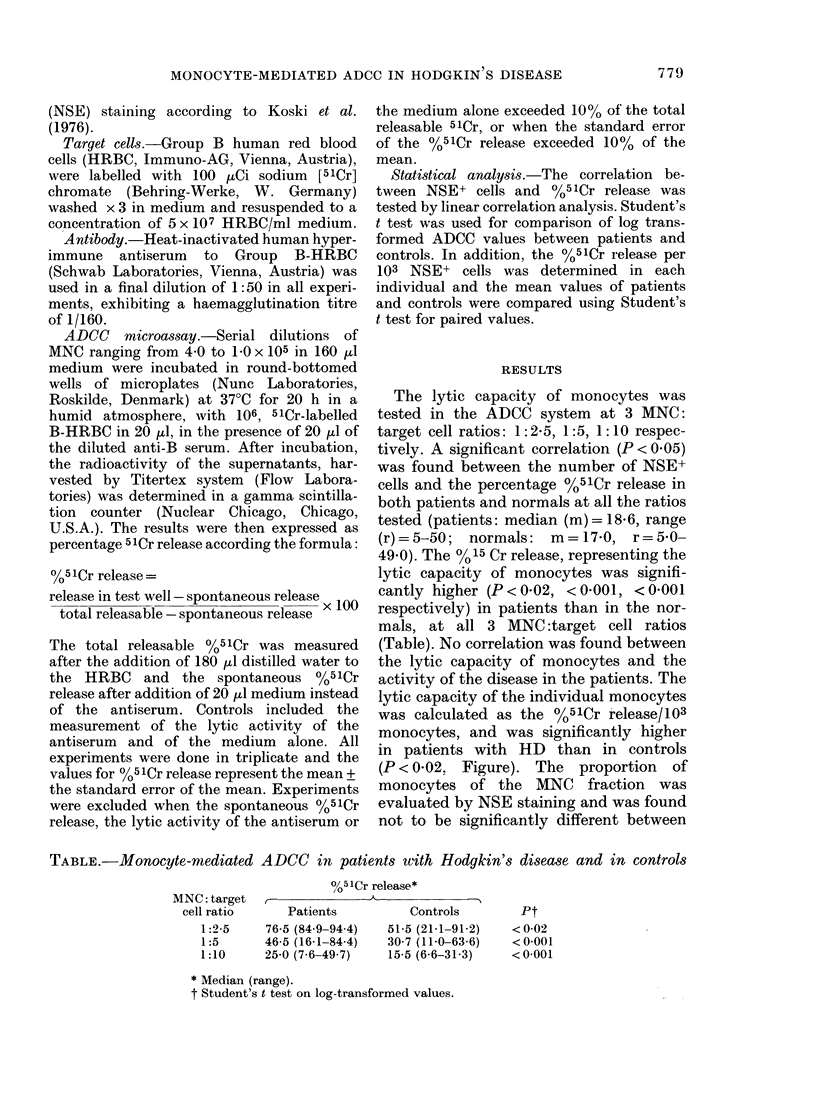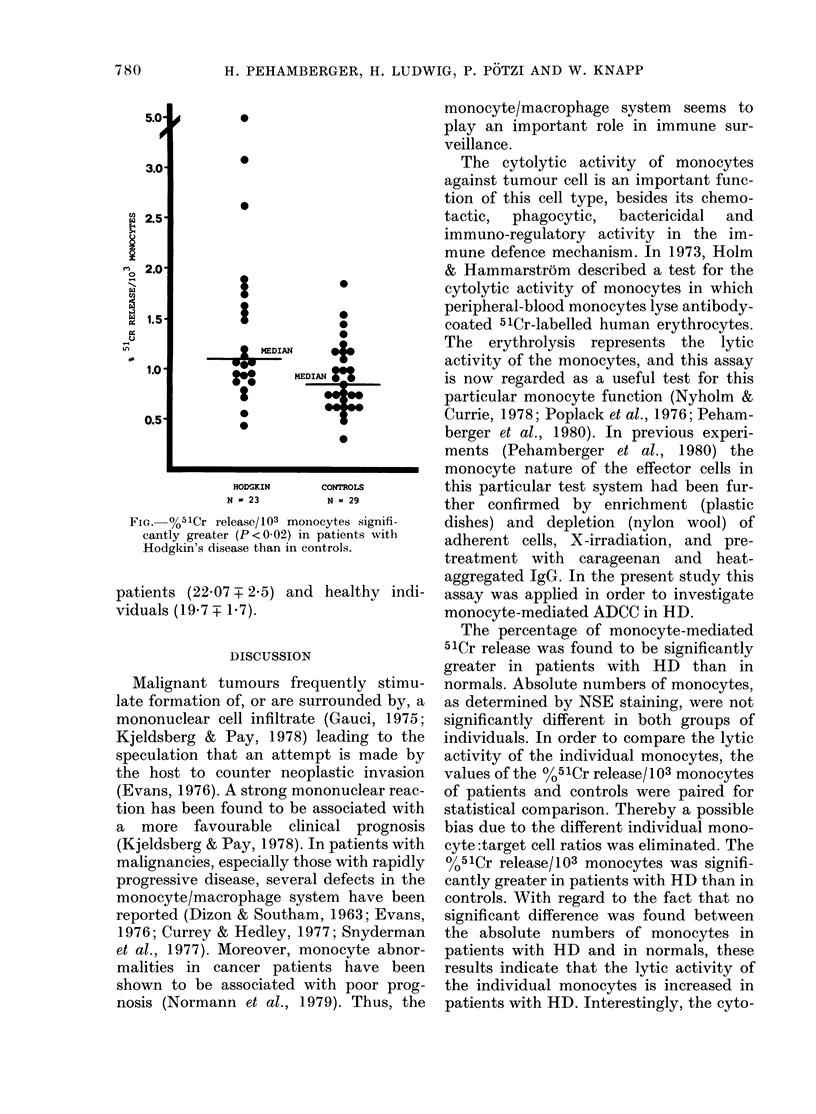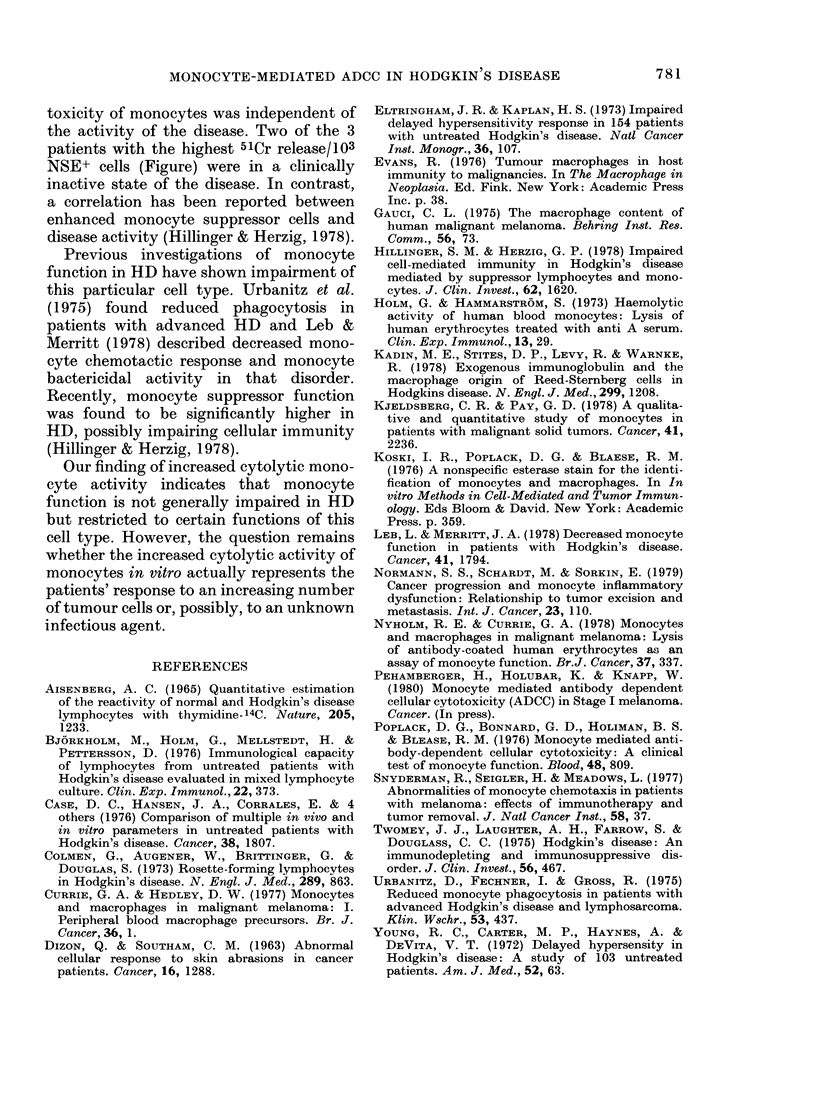# Increased monocyte-mediated antibody-dependent cellular cytotoxicity (ADCC) in Hodgkin's disease.

**DOI:** 10.1038/bjc.1980.141

**Published:** 1980-05

**Authors:** H. Pehamberger, H. Ludwig, P. Pötzi, W. Knapp

## Abstract

Monocyte-mediated antibody-dependent cellular cytotoxicity (ADCC) was tested in 23 patients with histologically proven Hodgkin's disease and 29 healthy normal controls. Seven patients presented with active and 16 with inactive disease. The lytic capacity of the individual monocytes was significantly (P < 0.02) higher in patients with Hodgkin's disease than in normals. However, no significant difference was found between the numbers of monocytes in both groups of individuals, as determined by non-specific esterase staining. No correlation was found between the lytic capacity of monocytes and the activity of the disease.


Br. J. Cancer (1980) 41, 778

### INCREASED MONOCYTE-MEDIATED ANTIBODY-DEPENDENT CELLULAR CYTOTOXICITY (ADCC) IN HODGKIN'S DISEASE

H. PEHAMBERGER‡, H. LUDWIG*, P. PÖTZI* AND W. KNAPP†

*From the Departments of ‡Dermatology I and *Internal Medicine II, and the †Institute of Immunology, University of Vienna, Austria*

Received 13 November 1979 Accepted 24 January 1980

**Summary.—Monocyte-mediated antibody-dependent cellular cytotoxicity (ADCC) was tested in 23 patients with histologically proven Hodgkin's disease and 29 healthy normal controls. Seven patients presented with active and 16 with inactive disease. The lytic capacity of the individual monocytes was significantly ($P<0{\cdot}02$) higher in patients with Hodgkin's disease than in normals. However, no significant difference was found between the numbers of monocytes in both groups of individuals, as determined by non-specific esterase staining. No correlation was found between the lytic capacity of monocytes and the activity of the disease.**

PATIENTS with Hodgkin's disease (HD) frequently exhibit impaired cell-mediated immunity (Aisenberg, 1965). Recent investigations indicate an anergy to recall antigens, failure of dinitrochlorobenzole sensitization, impaired mitogen reactivity (Case *et al.*, 1976; Eltringham & Kaplan, 1973) and reduced proliferation in mixed lymphocyte cultures (Björkholm *et al.*, 1976; Twomey *et al.*, 1975). In addition, lymphopenia (Young *et al.*, 1972) and lower percentages of T lymphocytes (Colmen *et al.*, 1973) have been reported.

Data on monocyte function in HD are rare, though the Reed-Sternberg cell seems to be of macrophage origin (Kadin *et al.*, 1978). Reduced phagocytic activity of monocytes (Urbanitz *et al.*, 1975), decreased chemotactic response and monocyte bactericidal activity (Leb & Merritt, 1978) have been reported. We studied monocyte-mediated antibody-dependent cellular cytotoxicity (ADCC) in patients with HD in order to investigate a further parameter of monocyte function. Increased ADCC of monocytes was detected in HD by using a particular *in vitro* ADCC system, in which antibody-coated human erythrocytes are killed by peripheral-blood monocytes (Holm & Hammarström, 1973; Poplack *et al.*, 1976; Pehamberger *et al.*, 1980; Nyholm & Currie, 1978).

### MATERIALS AND METHODS

*Patients.*—23 patients (13 male, 10 female; age range 16–42 years, mean $32 \pm 6{\cdot}3$) with histologically proven HD were investigated. All patients exhibited Stage III or IV of the disease, 7 presenting with active and 16 with inactive disease. Since depression of monocyte function may occur shortly after drug ingestion (Leb & Merritt, 1978) care was taken that none of the patients had received any cytostatic treatment for the last 3 weeks before investigation. In addition, none of the patients had active intercurrent infections when the study was performed. 29 normal, sex- and age-matched healthy volunteers served as controls.

*Effector cells.*—Mononuclear cell (MNC) suspensions were obtained by Ficoll-Hypaque separation and were washed ×3 in RPMI 1640 (Flow Laboratories, Bonn, W. Germany), supplemented with 10% heat-inactivated foetal calf serum (Flow Laboratories). The proportion of monocytes in the MNC preparation was evaluated by non-specific esterase

Correspondence to: Dr Hubert Pehamberger, Department of Dermatology I, University of Vienna, Alserstrasse 4, A 1090 Vienna, Austria.

(NSE) staining according to Koski *et al.* (1976).

*Target cells.*—Group B human red blood cells (HRBC, Immuno-AG, Vienna, Austria), were labelled with 100 $\mu$Ci sodium [$^{51}$Cr] chromate (Behring-Werke, W. Germany) washed $\times 3$ in medium and resuspended to a concentration of $5 \times 10^7$ HRBC/ml medium.

*Antibody.*—Heat-inactivated human hyperimmune antiserum to Group B-HRBC (Schwab Laboratories, Vienna, Austria) was used in a final dilution of 1:50 in all experiments, exhibiting a haemagglutination titre of 1/160.

*ADCC microassay.*—Serial dilutions of MNC ranging from $4{\cdot}0$ to $1{\cdot}0 \times 10^5$ in 160 $\mu$l medium were incubated in round-bottomed wells of microplates (Nunc Laboratories, Roskilde, Denmark) at 37°C for 20 h in a humid atmosphere, with $10^6$, $^{51}$Cr-labelled B-HRBC in 20 $\mu$l, in the presence of 20 $\mu$l of the diluted anti-B serum. After incubation, the radioactivity of the supernatants, harvested by Titertex system (Flow Laboratories) was determined in a gamma scintillation counter (Nuclear Chicago, Chicago, U.S.A.). The results were then expressed as percentage $^{51}$Cr release according the formula:

$$\%^{51}\text{Cr release} = \frac{\text{release in test well} - \text{spontaneous release}}{\text{total releasable} - \text{spontaneous release}} \times 100$$

The total releasable $\%^{51}$Cr was measured after the addition of 180 $\mu$l distilled water to the HRBC and the spontaneous $\%^{51}$Cr release after addition of 20 $\mu$l medium instead of the antiserum. Controls included the measurement of the lytic activity of the antiserum and of the medium alone. All experiments were done in triplicate and the values for $\%^{51}$Cr release represent the mean $\pm$ the standard error of the mean. Experiments were excluded when the spontaneous $\%^{51}$Cr release, the lytic activity of the antiserum or the medium alone exceeded 10% of the total releasable $^{51}$Cr, or when the standard error of the $\%^{51}$Cr release exceeded 10% of the mean.

*Statistical analysis.*—The correlation between NSE$^+$ cells and $\%^{51}$Cr release was tested by linear correlation analysis. Student's *t* test was used for comparison of log transformed ADCC values between patients and controls. In addition, the $\%^{51}$Cr release per $10^3$ NSE$^+$ cells was determined in each individual and the mean values of patients and controls were compared using Student's *t* test for paired values.

### RESULTS

The lytic capacity of monocytes was tested in the ADCC system at 3 MNC: target cell ratios: 1:2·5, 1:5, 1:10 respectively. A significant correlation ($P < 0{\cdot}05$) was found between the number of NSE$^+$ cells and the percentage $\%^{51}$Cr release in both patients and normals at all the ratios tested (patients: median (m) = 18·6, range (r) = 5–50; normals: m = 17·0, r = 5·0–49·0). The $\%^{15}$ Cr release, representing the lytic capacity of monocytes was significantly higher ($P < 0{\cdot}02$, $< 0{\cdot}001$, $< 0{\cdot}001$ respectively) in patients than in the normals, at all 3 MNC:target cell ratios (Table). No correlation was found between the lytic capacity of monocytes and the activity of the disease in the patients. The lytic capacity of the individual monocytes was calculated as the $\%^{51}$Cr release/$10^3$ monocytes, and was significantly higher in patients with HD than in controls ($P < 0{\cdot}02$, Figure). The proportion of monocytes of the MNC fraction was evaluated by NSE staining and was found not to be significantly different between

TABLE.—*Monocyte-mediated ADCC in patients with Hodgkin's disease and in controls*

| MNC: target cell ratio | $\%^{51}$Cr release* Patients | $\%^{51}$Cr release* Controls | $P$† |
|---|---|---|---|
| 1:2·5 | 76·5 (84·9–94·4) | 51·5 (21·1–91·2) | <0·02 |
| 1:5 | 46·5 (16·1–84·4) | 30·7 (11·0–63·6) | <0·001 |
| 1:10 | 25·0 (7·6–49·7) | 15·5 (6·6–31·3) | <0·001 |

* Median (range).
† Student's *t* test on log-transformed values.

FIG.—%$^{51}$Cr release/$10^3$ monocytes significantly greater ($P < 0{\cdot}02$) in patients with Hodgkin's disease than in controls.

patients ($22{\cdot}07 \mp 2{\cdot}5$) and healthy individuals ($19{\cdot}7 \mp 1{\cdot}7$).

### DISCUSSION

Malignant tumours frequently stimulate formation of, or are surrounded by, a mononuclear cell infiltrate (Gauci, 1975; Kjeldsberg & Pay, 1978) leading to the speculation that an attempt is made by the host to counter neoplastic invasion (Evans, 1976). A strong mononuclear reaction has been found to be associated with a more favourable clinical prognosis (Kjeldsberg & Pay, 1978). In patients with malignancies, especially those with rapidly progressive disease, several defects in the monocyte/macrophage system have been reported (Dizon & Southam, 1963; Evans, 1976; Currey & Hedley, 1977; Snyderman *et al.*, 1977). Moreover, monocyte abnormalities in cancer patients have been shown to be associated with poor prognosis (Normann *et al.*, 1979). Thus, the monocyte/macrophage system seems to play an important role in immune surveillance.

The cytolytic activity of monocytes against tumour cell is an important function of this cell type, besides its chemotactic, phagocytic, bactericidal and immuno-regulatory activity in the immune defence mechanism. In 1973, Holm & Hammarström described a test for the cytolytic activity of monocytes in which peripheral-blood monocytes lyse antibody-coated $^{51}$Cr-labelled human erythrocytes. The erythrolysis represents the lytic activity of the monocytes, and this assay is now regarded as a useful test for this particular monocyte function (Nyholm & Currie, 1978; Poplack *et al.*, 1976; Pehamberger *et al.*, 1980). In previous experiments (Pehamberger *et al.*, 1980) the monocyte nature of the effector cells in this particular test system had been further confirmed by enrichment (plastic dishes) and depletion (nylon wool) of adherent cells, X-irradiation, and pretreatment with carageenan and heat-aggregated IgG. In the present study this assay was applied in order to investigate monocyte-mediated ADCC in HD.

The percentage of monocyte-mediated $^{51}$Cr release was found to be significantly greater in patients with HD than in normals. Absolute numbers of monocytes, as determined by NSE staining, were not significantly different in both groups of individuals. In order to compare the lytic activity of the individual monocytes, the values of the %$^{51}$Cr release/$10^3$ monocytes of patients and controls were paired for statistical comparison. Thereby a possible bias due to the different individual monocyte:target cell ratios was eliminated. The %$^{51}$Cr release/$10^3$ monocytes was significantly greater in patients with HD than in controls. With regard to the fact that no significant difference was found between the absolute numbers of monocytes in patients with HD and in normals, these results indicate that the lytic activity of the individual monocytes is increased in patients with HD. Interestingly, the cytotoxicity of monocytes was independent of the activity of the disease. Two of the 3 patients with the highest $^{51}$Cr release/$10^3$ NSE$^+$ cells (Figure) were in a clinically inactive state of the disease. In contrast, a correlation has been reported between enhanced monocyte suppressor cells and disease activity (Hillinger & Herzig, 1978).

Previous investigations of monocyte function in HD have shown impairment of this particular cell type. Urbanitz *et al.* (1975) found reduced phagocytosis in patients with advanced HD and Leb & Merritt (1978) described decreased monocyte chemotactic response and monocyte bactericidal activity in that disorder. Recently, monocyte suppressor function was found to be significantly higher in HD, possibly impairing cellular immunity (Hillinger & Herzig, 1978).

Our finding of increased cytolytic monocyte activity indicates that monocyte function is not generally impaired in HD but restricted to certain functions of this cell type. However, the question remains whether the increased cytolytic activity of monocytes *in vitro* actually represents the patients' response to an increasing number of tumour cells or, possibly, to an unknown infectious agent.

### REFERENCES

AISENBERG, A. C. (1965) Quantitative estimation of the reactivity of normal and Hodgkin's disease lymphocytes with thymidine-$^{14}$C. *Nature*, **205**, 1233.

BJÖRKHOLM, M., HOLM, G., MELLSTEDT, H. & PETTERSSON, D. (1976) Immunological capacity of lymphocytes from untreated patients with Hodgkin's disease evaluated in mixed lymphocyte culture. *Clin. Exp. Immunol.*, **22**, 373.

CASE, D. C., HANSEN, J. A., CORRALES, E. & 4 others (1976) Comparison of multiple *in vivo* and *in vitro* parameters in untreated patients with Hodgkin's disease. *Cancer*, **38**, 1807.

COLMEN, G., AUGENER, W., BRITTINGER, G. & DOUGLAS, S. (1973) Rosette-forming lymphocytes in Hodgkin's disease. *N. Engl. J. Med.*, **289**, 863.

CURRIE, G. A. & HEDLEY, D. W. (1977) Monocytes and macrophages in malignant melanoma: I. Peripheral blood macrophage precursors. *Br. J. Cancer*, **36**, 1.

DIZON, Q. & SOUTHAM, C. M. (1963) Abnormal cellular response to skin abrasions in cancer patients. *Cancer*, **16**, 1288.

ELTRINGHAM, J. R. & KAPLAN, H. S. (1973) Impaired delayed hypersensitivity response in 154 patients with untreated Hodgkin's disease. *Natl Cancer Inst. Monogr.*, **36**, 107.

EVANS, R. (1976) Tumour macrophages in host immunity to malignancies. In *The Macrophage in Neoplasia*. Ed. Fink. New York: Academic Press Inc. p. 38.

GAUCI, C. L. (1975) The macrophage content of human malignant melanoma. *Behring Inst. Res. Comm.*, **56**, 73.

HILLINGER, S. M. & HERZIG, G. P. (1978) Impaired cell-mediated immunity in Hodgkin's disease mediated by suppressor lymphocytes and monocytes. *J. Clin. Invest.*, **62**, 1620.

HOLM, G. & HAMMARSTRÖM, S. (1973) Haemolytic activity of human blood monocytes: Lysis of human erythrocytes treated with anti A serum. *Clin. Exp. Immunol.*, **13**, 29.

KADIN, M. E., STITES, D. P., LEVY, R. & WARNKE, R. (1978) Exogenous immunoglobulin and the macrophage origin of Reed-Sternberg cells in Hodgkins disease. *N. Engl. J. Med.*, **299**, 1208.

KJELDSBERG, C. R. & PAY, G. D. (1978) A qualitative and quantitative study of monocytes in patients with malignant solid tumors. *Cancer*, **41**, 2236.

KOSKI, I. R., POPLACK, D. G. & BLAESE, R. M. (1976) A nonspecific esterase stain for the identification of monocytes and macrophages. In *In vitro Methods in Cell-Mediated and Tumor Immunology*. Eds Bloom & David. New York: Academic Press. p. 359.

LEB, L. & MERRITT, J. A. (1978) Decreased monocyte function in patients with Hodgkin's disease. *Cancer*, **41**, 1794.

NORMANN, S. S., SCHARDT, M. & SORKIN, E. (1979) Cancer progression and monocyte inflammatory dysfunction: Relationship to tumor excision and metastasis. *Int. J. Cancer*, **23**, 110.

NYHOLM, R. E. & CURRIE, G. A. (1978) Monocytes and macrophages in malignant melanoma: Lysis of antibody-coated human erythrocytes as an assay of monocyte function. *Br.J. Cancer*, **37**, 337.

PEHAMBERGER, H., HOLUBAR, K. & KNAPP, W. (1980) Monocyte mediated antibody dependent cellular cytotoxicity (ADCC) in Stage I melanoma. *Cancer*. (In press).

POPLACK, D. G., BONNARD, G. D., HOLIMAN, B. S. & BLEASE, R. M. (1976) Monocyte mediated antibody-dependent cellular cytotoxicity: A clinical test of monocyte function. *Blood*, **48**, 809.

SNYDERMAN, R., SEIGLER, H. & MEADOWS, L. (1977) Abnormalities of monocyte chemotaxis in patients with melanoma: effects of immunotherapy and tumor removal. *J. Natl Cancer Inst.*, **58**, 37.

TWOMEY, J. J., LAUGHTER, A. H., FARROW, S. & DOUGLASS, C. C. (1975) Hodgkin's disease: An immunodepleting and immunosuppressive disorder. *J. Clin. Invest.*, **56**, 467.

URBANITZ, D., FECHNER, I. & GROSS, R. (1975) Reduced monocyte phagocytosis in patients with advanced Hodgkin's disease and lymphosarcoma. *Klin. Wschr.*, **53**, 437.

YOUNG, R. C., CARTER, M. P., HAYNES, A. & DEVITA, V. T. (1972) Delayed hypersensity in Hodgkin's disease: A study of 103 untreated patients. *Am. J. Med.*, **52**, 63.